# Effects of the Interactions between Dust Exposure and Genetic Polymorphisms in Nalp3, Caspase-1, and IL-1β on the Risk of Silicosis: A Case-Control Study

**DOI:** 10.1371/journal.pone.0140952

**Published:** 2015-10-23

**Authors:** Shaofan Weng, Lihua Wang, Yi Rong, Yuewei Liu, Xin Wang, Hongyu Guan, Weihong Chen

**Affiliations:** 1 Department of Occupational and Environmental Health, School of Public Health, Tongji Medical College, Huazhong University of Science and Technology, Wuhan, Hubei, China; 2 Shenzhen Prevention and Treatment Center for Occupational Disease, Shenzhen, Guangdong, China; 3 Shenzhen Baoan Center for Disease Control and Prevention, Shenzhen, Guangdong, China; 4 Daye Iron Mine Hospital, Wuhan Iron and Steel Corporation, Huangshi, Hubei, China; institute of Biosciences and and BioResources, ITALY

## Abstract

**Objectives:**

To evaluate the effects of the interactions between polymorphisms in Nalp3, caspase-1, and interleukin(IL)-1β genes and occupational dust exposure on the risk of silicosis.

**Methods:**

We conducted a population-based case-control study in a large iron mine in China. Between January 2006 and December 2009, we identified 179 patients with silicosis to evaluate as cases and 201 individuals without silicosis to evaluate as controls. We estimated cumulative dust exposure (CDE) for all subjects and we genotyped polymorphisms in Nalp3, caspase-1, and IL-1β genes. We estimated odds ratios(ORs), 95% confidence intervals(95%CIs), and *p*-values using logistic regression models adjusted for selected confounders.

**Results:**

After adjusting for age, smoking status, and CDE, subjects with the CT genotype of Ex4-849C>T in Nalp3 and the GA genotype of Ex2+37G>A in caspase-1 had increased risks of silicosis (adjusted ORs[95%CIs] = 2.40 [1.12–5.12] and 3.62 [1.63–8.02], respectively). Among subjects younger than 70 years old, those with the CC genotype of IVS8-7652A>C in Nalp3 had a lower risk of silicosis than those with other genotypes (adjusted OR[95%CI] = 0.24[0.06–0.88]). Among subjects aged 70 years and older, those with the CT genotype of Ex4-849C>T in Nalp3 and those with the GA genotype of Ex2+37G>A in caspase-1 had a higher risk of silicosis than those with other genotypes (adjusted ORs [95%CI] = 2.52[1.04–6.12] and 5.19[1.88–14.35], respectively). Among subjects with CDE greater than 120 mg/m^3^×year and among smokers, those with the GA genotype of Ex2+37G>A in caspase-1 had a higher risk of silicosis than those with other genotypes (adjusted ORs[95%CIs] = 26.37[3.35–207.39] and 3.47[1.40–8.64], respectively).

**Conclusions:**

Genetic polymorphisms in Nalp3 and caspase-1 may be associated with individual susceptibility to silicosis, especially when the polymorphisms interact with age, CDE, or smoking status.

## Introduction

Silicosis is an occupational fibrotic lung disease that is induced by the inhalation and deposition of free crystalline silica dust[1]. Crystalline silica dust exposure is one of the oldest and most serious occupational hazards worldwide and occurs primarily in industries such as metals or coal mining, construction, and glass and clay manufacturing[2]. The United States Occupational Safety and Health Administration estimated that 2.2 million American workers were exposed to silica dust in 2003[3]. In China and India, estimates of silica exposure are much higher and reports indicate that more than 23 million and more than 10 million workers, respectively, are exposed to silica dust in those countries. In Europe, estimates are comparable to those of the United States, with at least 2 million workers exposed to silica dust[4,5,6,7]. The incidence of silicosis has decreased significantly in developed countries in recent decades due to the control of silica exposure, but silicosis remains a substantial public health concern in developing countries. In a cohort study of 49,309 Chinese workers, we observed that the cumulative incidence of silicosis was 22.3% among workers exposed to silica dust[7]. In addition to cumulative silica dust exposure, other factors, including genetic susceptibility, have been implicated in the development of silicosis. Cytokines such as transforming growth factor (TGF)-β1, interleukin (IL)-1β, IL-1α, IL-4, IL-6, and IL-13 have been suggested to play important roles during the early inflammatory response in pneumoconiosis[8,9,10]. The severity of pulmonary fibrosis varies greatly among workers in the same work environment, which suggests that genetic variants of the genes involved in key pathological processes may influence the development and progression of silicosis [11].

Current evidence from clinical studies and experimental animal models suggests that chronic pulmonary inflammation is an essential process in the pathogenesis of silicosis[12,13]. Recent studies have found that silica could be sensed by the Nalp3 inflammasome; the activation of Nalp3 then leads to IL-1β secretion and persistent chronic inflammation[14,15].

The Nalp3 inflammasome is an intracellular, multiprotein complex composed of Nalp3, at least one adaptor protein, and caspase-1, which is an IL-1β-cleaving enzyme[16]. Once activated, the Nalp3 inflammasome activates caspase-1 and then cleaves inactive IL-1β to form active IL-1β, which is secreted by cells and binds to the IL-1 receptor to initiate inflammation[17]. In silicosis, IL-1β has been implicated to participate in the processes of silica-induced pulmonary inflammation and fibrosis[18,19]. Therefore, we hypothesized that genetic polymorphisms in Nalp3, caspase-1, and IL-1β may affect an individual’s susceptibility to silicosis.

In order to investigate the interactions of genetic polymorphisms of Nalp3, caspase-1, and IL-1β with occupational dust exposure and the joint effects on the risk of silicosis, we conducted a case-control study of a large cohort of workers in a large iron mine. Individual dust exposure was estimated by work history and a dust exposure matrix. We examined the variant frequencies and genotype distributions of the 5 SNPs for each of the subjects and compared these distributions to the presence of silicosis.

## Materials and Methods

### Study subjects

Between January 2006 and December 2009, we identified 179 patients with silicosis and 201 individuals without silicosis from an iron mine that belongs to Wuhan Iron and Steel Corporation located in Huangshi City, Hubei Province, China. All workers that were exposed to silica received chest radiographs every 2 to 4 years. These test results and medical records, including date and stage of silicosis diagnosis and progression, were maintained by the mine hospital. The cases of silicosis were diagnosed by at least 3 qualified physicians according to China National Criteria for silicosis, which is consistent with International Labor Organization Classification[20,21]. We frequency-matched the control subjects to the silicosis cases by date of birth (±3 years), year of starting work, and job type. In the mine we investigated, only 3 female workers and 1 non-Han ethnic worker had been diagnosed with silicosis. To avoid effects or interactions related to sex or ethnic group, we excluded these workers from the final analysis.

Trained investigators used a questionnaire to conduct a face-to-face interview to collect general information on age, lifestyle (e.g., cigarette smoking, alcohol consumption, and fuel used for cooking), and medical history (e.g., stage and date of diagnosis of silicosis and the presence of other respiratory diseases such as pneumonia and chronic obstructive pulmonary disease) from each of the subjects. Medical histories were verified by reviewing health profiles saved by the mine hospital. Complete work histories for each subject were obtained from personal employment records in the mine’s files. Work histories included all job titles as well as the starting and ending years for each worker’s full duration of employment. More than 7800 environmental measurements of total dust concentration for different job titles were collected from 1953 to 2009; we used these measurements to create a job-exposure matrix. We used this matrix and occupational histories to calculate the CDE (mg/m^3^×year) for each subject. Detailed dust exposure estimations have been described in previous reports[7,20].

Each study subject provided written informed consent for the interview and blood sample collection. The study was approved by the Tongji Medical College Institutional Review Board and was conducted according to all current ethical guidelines. A 5-ml venous blood sample was collected from each subject at enrollment and stored at -80°C in a collection tube with 2% EDTA.

We investigated 3 candidate genes (Nalp3, caspase-1, and IL-1β) that are proposed to affect inflammatory and fibrosis processes. We selected 5 single nucleotide polymorphisms (SNPs) among the 3 genes on the basis of their minor allele frequency (MAF) and potential biological function. From the HapMap Project database (www.hapmap.org), we selected 2 tagging SNPs using the SNP browser (Applied Biosystems, Foster City, CA, USA; www.allsnps.com/snpbrowser) with a cut-off of *r* ^2^ = 0.8 and an MAF ≥10% in white people and Chinese populations. For comprehensive coverage of the polymorphisms, we chose 3 additional SNPs from the coding region (nonsynonymous or synonymous), untranslated region, promoter region, or splicing sites with an MAF ≥ 2.5% in Chinese populations. Table 1 summarizes the genes, nucleotide substitutions, functions (e.g., encoding amino acid changes), reference SNP identification numbers, and reported allele frequencies of the SNPs evaluated in this study. The mRNA transcripts, protein sequences, structures, homology models, and predicted functions for the SNPs were evaluated by F-SNP software (Queen’s University, Kingston, Ontario, Canada).

**Table 1:** Summary of the single nucleotide polymorphisms(SNPs) among the 3 candidate genes.

Gene	Chromosome	RS#	SNP	Function	MAF
NLRP3	1q44	rs1539019	IVS8-7652A>C	Intron	0.43
		rs34298354	Ex4-849C>T	S436S	0.027
CASP1	11q23	rs1042743	Ex2+37G>A	R15H	0.057
IL1B	2q14	rs1143634	Ex5+14C>T	F105F	0.037
		rs1143627	-580C>T	promoter	0.45

MAF, minor allele frequency.

RS#, reference SNP identification number.

### DNA preparation and genotyping assays

We extracted genomic DNA from 300-μL blood samples using a DNA purification kit (Gentra System, Minneapolis, MN, USA) according to the manufacturer’s instructions. Subsequent genotyping for all subjects was conducted with the TaqMan method using a 384-well format on the ABI 7900HT Real Time polymerase chain reaction (PCR) system (Applied Biosystems) according to the manufacturer’s instructions. The TaqMan Assay kit (Applied Biosystems) included PCR primers (forward and reverse) and the TaqMan MGB probes labeled with 2 dyes (FAM and VIC). PCR reactions were completed in a reaction volume of 5μL, which contained 5ng DNA, 2.5μL 2X TaqMan Universal PCR Master Mix without AmpErase UNG (Applied Biosystems), and 0.125μL 40X Assay Mix (Applied Biosystems). PCR conditions were as follows: 10 min at 95°C, 40 cycles of 15s at 92°C, and 1 min at 60°C. To ensure the quality of the experiment, approximately 10% of the samples were repeated; the concordance was 100%. The intensity of each SNP met the criteria of 3 clear clusters in 2 scales generated by the Sequence Detection Systems software, version 2.2.1 (Applied Biosystems).

Briefly, the PCR primers were designed as follows: IVS8-7652A>C in Nalp3, 5’-AAAATAAAATTCAGGGAGCCTCATA-3’(forward) and 5’-CTTTGTCCAAGGACACTCAGAGAGA-3’(reverse); Ex4-849C>T in Nalp3, 5’-CACTGGGTCATGTTTAATTTCC-3’(forward) and 5’-ATGTGTTTGTATAGTTTCCAGAATT-3’(reverse); Ex2+37G>A in caspase-1, 5’-ATTTATTGTACCTTCACCCATGGAA-3’(forward) and 5’-GGATAAACAGCTTTCTCTTCTCCTT-3’(reverse); Ex5+14C>T in IL-1β, 5’-CATAAGCCTCGTTATCCCATGTGTC-3’(forward) and 5’-AAGAAGATAGGTTCTGAAATGTGGA-3’(reverse); -580C>T in IL-1β, 5’-CCAGTTTCTCCCTCGCTGTTTTTAT-3’(forward) and 5’-GCTTTCAAAAGCAGAAGTAGGAGGC-3’(reverse).

### Statistical analysis

Differences in the distributions of demographic characteristics and frequencies of genotypes of Nalp3, caspase-1, and IL-1β polymorphisms among silicosis cases and controls were evaluated using the Student’s t-test or the χ^2^ test. Associations between SNPs and the presence of silicosis were estimated using logistic regression models that were adjusted for selected confounders. A *p*-value<0.05 indicated statistical significance. All analyses were performed using SAS software, version 9.1(SAS Institute, Cary, NC, USA).

## Results

The lifestyle and occupational characteristics of patients with silicosis and control subjects are listed in Table 2. There were no differences between the case and control groups in smoking status, smoking years, pack-years, or average age. Although the CDE was similar, the average number of years of dust exposure of silicosis patients was shorter than that of the controls (*p*<0.0001). This finding is due, in part, to the fact that workers were required to transfer to non-dust exposure jobs once silicosis was diagnosed.

**Table 2:** Lifestyle and occupational characteristics of patients with silicosis and control subjects.

Variable	Cases (n = 179)	Controls (n = 201)	*p* value^a^
Smoking status^b^, n (%)			0.5489
No	45(25.14)	56 (27.86)	
Yes	134 (74.86)	145 (72.14)	
Smoking years^c^	42.26±12.50	39.86±10.25	0.0799
Pack-years^c^	21.45±11.18	22.28±11.90	0.4656
Dust exposure years	22.38±8.10	26.17±9.86	<0.0001
CDE	121.79±63.52	129.54±69.51	0.2593
Average age(years)	73.21±6.34	73.09±5.90	0.8480

^a^ All tests were 2-sided.

^b^Former smokers and current smokers were categorized as having a smoking status of “yes.”

^c^Subjects who never smoked were excluded from the calculation.

CDE, cumulative dust exposure.

Table 3 lists the genotype frequencies among silicosis cases and controls. No significant differences were found in the genotype frequencies of the IVS8-7652A>C polymorphism in Nalp3 between patients with silicosis and subjects in the control group. The distribution of the Ex4-849C>T polymorphism in Nalp3 was significantly different between the cases and controls (p = 0.0185). After adjusting for age, smoking status, and CDE, subjects with the T allele had a significantly increased risk of silicosis compared to those with the C allele (OR [95%CI] = 2.40[1.12–5.12]). The distributions of -580C>T and Ex5+14C>T polymorphisms in IL-1β were not different between patients with silicosis and subjects in control group. After adjusting for age, smoking status, and CDE, the frequency of the GA genotype for the Ex2+37G>A polymorphism in caspase-1 was significantly higher among silicosis cases than controls (OR [95%CI] = 3.62 [1.63–8.02]).

**Table 3:** Genotype frequencies and associated risks of silicosis in case and control subjects.

Variables	Cases(No, %)	Controls(No, %)	Crude OR (95%CI)	Adjusted OR(95%CI)^a^	*p* value
Nalp3					
IVS8-7652A>C					
AA	70(39.1)	70(34.8)	1.00	1.00	
AC	80(44.7)	93(46.3)	0.86 (0.55–1.34)	0.86 (0.55–1.35)	0.9685
CC	29(16.2)	38(18.9)	0.77 (0.43–1.37)	0.76 (0.42–1.36)	0.4531
Ex4-849C>T					
CC	157(87.7)	190(94.5)	1.00	1.00	
CT	22(12.3)	11(5.5)	2.42 (1.14–5.14)	2.40 (1.12–5.12)	0.0239
IL-1β					
-580C>T					
CC	55(30.7)	54(26.9)	1.00	1.00	
CT	91(50.8)	100(49.8)	0.89 (0.56–1.43)	0.87 (0.55–1.40)	0.8133
TT	33(18.4)	47(23.4)	0.69 (0.39–1.23)	0.68 (0.38–1.22)	0.2370
Ex5+14C>T					
CC	173(96.6)	188(93.5)	1.00	1.00	
CT	6(3.4)	13(6.5)	0.50 (0.19–1.35)	0.52 (0.19–1.40)	0.1946
Caspase-1					
Ex2+37G>A					
GG	154(86.0)	192(95.5)	1.00	1.00	
GA	25(14.0)	9(4.5)	3.46 (1.57–7.64)	3.62 (1.63–8.02)	0.0038

^a^ Data were calculated using unconditional logistic regression and adjusting for age, smoking status, and CDE.

CI, confidence interval; OR, odds ratio.

The results of our stratification analysis for the SNPs of Nalp3 are presented in Table 4. Among subjects younger than 70 years old, individuals with the CC genotype of IVS8-7652A>C in Nalp3 had a lower risk of silicosis than individuals with other genotypes (OR [95%CI] = 0.24[0.06–0.88]). Among subjects older than 70 years, individuals with the T allele of Ex4-849C>T in Nalp3 had a higher risk of silicosis than those with other genotypes (OR [95%CI] = 2.52 [1.04–6.12]); the T allele was also associated with a higher risk of silicosis in smokers (OR [95%CI] = 2.57 [1.11–5.93]).

**Table 4:** Stratification analysis for associations between genotypes of Nalp3 and the risk of silicosis.

	IVS8 -7652A>C OR(95%CI)^*^			Ex4-849C>T OR(95%CI) ^*^	
	AA	AC	CC	CC	CT
CDE(mg/m^3^-y)					
<120	1.00	1.16(0.62–2.16)	1.12(0.47–2.66)	1.00	2.06(0.78–5.45)
≥120	1.00	0.60(0.31–1.19)	0.45(0.19–1.05)	1.00	3.05(0.88–10.66)
Age(years)					
<70	1.00	1.28(0.48–3.42)	0.24(0.06–0.88)	1.00	1.69(0.36–7.85)
≥70	1.00	0.77(0.46–1.29)	1.09(0.54 to 2.18)	1.00	2.52(1.04–6.12)
Smoking					
No	1.00	1.05(0.43–2.55)	1.59(0.49–5.15)	1.00	1.63(0.25–10.49)
Yes	1.00	0.82(0.49–1.39)	0.61(0.31–1.22)	1.00	2.57(1.11–5.93)

^*^ORs were obtained from a logistic regression model that was adjusted for age, smoking status, and CDE.

The results of our stratification analysis for the SNPs of IL-1β are presented in Table 5. We did not observe any significant interactions between the 2 SNPs in IL-1β and CDE, age, or smoking status.

**Table 5:** Stratification analysis for associations between genotypes of IL-1β and the risk of silicosis.

	-580C>T OR (95%CI)^*^			Ex5+14C>T OR (95%CI)^*^	
	CC	CT	TT	CC	CT
CDE(mg/m^3^-y)					
<120	1.00	0.93(0.47–1.84)	0.56(0.24–1.27)	1.00	0.14(0.02–1.14)
≥120	1.00	0.96(0.48–1.92)	0.56(0.24–1.31)	1.00	0.96(0.27–3.40)
Age(years)					
<70	1.00	0.60(0.22–1.63)	0.58(0.17–2.02)	1.00	—
≥70	1.00	1.00(0.58–1.73)	0.76(0.39–1.49)	1.00	0.54(0.20–1.48)
Smoking					
No	1.00	1.32(0.52–3.38)	1.22(0.40–3.74)	1.00	0.25(0.03–2.24)
Yes	1.00	0.78(0.45–1.36)	0.57(0.28–1.13)	1.00	0.66(0.21–2.07)

^*^ORs were obtained from a logistic regression model that was adjusted for age, smoking status, and CDE.

The results of our stratification analysis for the SNPs of caspase-1 are presented in Table 6. Individuals with the GA genotype of Ex2+37G>A in caspase-1 had a higher risk of silicosis than individuals with the GG genotype if they had a CDE greater than 120 mg/m^3^×years (OR [95%CI] = 26.37 [3.35–207.39]), were older than 70 years old (OR [95%CI] = 5.19 [1.88–14.35]), or were smokers (OR [95%CI] = 3.47 [1.40–8.64]).

**Table 6:** Stratification analysis for associations between genotypes of caspase-1 and the risk of silicosis.

	Ex2+37G>A OR (95%CI)^*^	
	GG	GA
CDE (mg/m^3^-y)		
<120	1.00	1.04(0.37–2.91)
≥120	1.00	26.37(3.35–207.39)
Age (years)		
<70	1.00	1.36(0.33–5.60)
≥70	1.00	5.19(1.88–14.35)
Smoking		
No	1.00	3.80(0.72–20.11)
Yes	1.00	3.47(1.40–8.64)

^*^ORs were obtained from logistic regression models that were adjusted for age, smoking status, and CDE.

## Discussion

In this case-control study, 5 SNPs in Nalp3, IL-1β, and caspase-1 genes were evaluated to define the risk of silicosis in a Chinese population. We found 2 SNPs (Ex4-849C>T in Nalp3 and Ex2+37G>A in caspase-1) that were significantly associated with an increased risk of silicosis. The risk of silicosis was greater when CDE was greater than 120 mg/m^3^×years.

Silica is one of the most ubiquitous minerals on the earth and occupational silica exposure is very common. Inhalation of silica through occupational exposures may result in silicosis, which is one of the most important occupational diseases worldwide[22]. In Europe, more than 3 million workers were exposed to silica at work from 1990 to 1993[23]. Recent estimates of silica exposure in China are much higher and indicate that more than 23 million workers were exposed to silica in China in 2008[6,7].

Previous studies have shown that the incidence of silicosis may be influenced by genetic factors such as IL-4, IL-1, and tumor necrosis factor (TNF)-α[9,10,24]. Wang et al. assessed the association of TNF-α and IL-1RA SNPs with the risk of silicosis, and the results suggested that a polymorphism of IL-1RA (+2018) is associated with the risk of silicosis[24]. IL-1 is known to be a strong pro-inflammatory cytokine and the IL-1 signaling pathway plays a crucial role in subsequent inflammation and fibrosis[25]. IL-1β is synthesized as an inactive biological precursor that requires cleavage by cysteine protease caspase-1 for functional activity[26]. Caspase-1 itself is synthesized as an inactivate precursor[27] and the Nalp3 inflammasome can activate caspase-1.

The Nalp3 inflammasome is essential for the inflammatory response and subsequent development of pulmonary fibrosis after the inhalation of silica. Polymorphisms in Nalp3 that lead to amino acid changes have been described in Crohn’s disease, cardiovascular disease, and human immunodeficiency virus-1 infection[28,29,30]. We found that the T allele of Ex4-849C>T in Nalp3 was associated with a significantly increased risk of silicosis in Chinese iron miners, especially in persons of older age and in smokers. Population-based and animal studies have shown that smoking is associated with lung fibrosis[31,32], which is consistent with our findings. IVS8-7652A>C in Nalp3 has been shown to be associated with increased fibrinogen levels in blood[28]. In our study, the CC genotype was associated with a lower risk of silicosis only in subjects younger than 70 years old. However, since our sample size was relatively small, the relationship between IVS8-7652A>C polymorphisms in Nalp3 and the risk of silicosis needs further investigation in a larger population.

Caspase-1, the first identified caspase, is involved in the processing and secretion of pro-inflammatory molecules[33]. Recently, several studies explored the functions of caspase-1 in respiratory diseases[34,35]. In mouse models of cigarette smoke-induced pulmonary inflammation, caspase-1 knockout mice had significantly lower levels of IL-1β compared to wild-type mice[36], which suggests that caspase-1 is critical in pulmonary inflammation. More importantly, inflammation is a hallmark in the progression of silicosis[37]. In the present study, we evaluated the relationship between the Ex2+37G>A polymorphism in caspase-1 and the risk of silicosis in Chinese iron miners. Our results showed that the minor allele A of Ex2+37G>A in caspase-1 was associated with a significantly increased risk of silicosis.

IL-1β is known to be a very strong pro-inflammatory cytokine and it has been implicated in inflammatory disorders[17]. Some studies have shown that IL-1β polymorphisms are associated with type 2 diabetes and chronic periodontitis[38,39]. In patients with cystic fibrosis lung disease, IL-1β polymorphisms are associated with disease severity and lung function[40]. Inflammation and fibrosis are critical steps in the progression of silicosis. IL-1β has been implicated in the deposition of collagen, and the IL-1 receptor antagonist may be able to reduce pulmonary fibrosis provoked by silica and bleomycin[41]. A previous study conducted by our research group found that the neutralization of IL-1β attenuated silica-induced fibrosis by inhibiting the gene expression of TGF-β1, collagen I, and fibronectin. Elevated IL-1β may increase collagen expression[19,42]. In this study, we evaluated 2 SNPs in IL-1β (-580C>T and Ex5+14C>T) and their relationships with silicosis. However, the variants of IL-1β were not significantly associated with silicosis. Yucesoy et al. reported that SNPs in IL-1 receptor antagonist and TNF-α genes were associated with silicosis in Caucasian miners, but SNPs in IL-1α and IL-1β genes were not associated with the risk of silicosis[43,44]. These findings are similar to those of our current study. Ji et al. investigated associations between SNPs in inflammasome genes and coal workers’ pneumoconiosis (CWP) and found that the NLRP3 rs1539019 polymorphism may be associated with an increased risk for the development of CWP[45]. This finding differs from that of our study. However, the sample size in our study is relatively small, so we cannot exclude the potential contributions of -580C>T and Ex5+14C>T on the risk of silicosis. A larger sample size is needed for further investigation.

In conclusion, we found 2 SNPs (Ex4-849C>T of Nalp3 and Ex2+37G>A of caspase-1) that are associated with an increased risk of silicosis in a Chinese population. Further validation studies with larger, diverse populations are warranted to confirm our findings.
